# Editorial: Developments in Campylobacter, Helicobacter & Related Organisms Research – CHRO 2019

**DOI:** 10.3389/fmicb.2020.622582

**Published:** 2021-01-08

**Authors:** Nicolae Corcionivoschi, Stuart A. Thompson, Ozan Gundogdu

**Affiliations:** ^1^Bacteriology Branch, Veterinary Sciences Division, Agri-Food and Biosciences Institute (AFBI), Belfast, United Kingdom; ^2^Medical College of Georgia, Augusta University, Augusta, GA, United States; ^3^Department of Infection Biology, Faculty of Infectious and Tropical Diseases, London School of Hygiene and Tropical Medicine, London, United Kingdom

**Keywords:** *Campylobacter*, *Helicobacter*, CHRO 2019, gastrointestinal pathogen, *Campylobacter Helicobacter* and Related Organisms

*Campylobacter* spp. and *Helicobacter* spp. are important gastrointestinal pathogens that are major causes of acute gastroenteritis and gastric disease, respectively (Polk and Peek, 2010; Gundogdu and Wren, 2020). *Campylobacter* spp. are considered the leading bacterial cause of human gastroenteritis. In low-resource settings, *Campylobacter* infections are common in young children and correlate with stunted growth and life-long physical and cognitive deficiencies (Amour et al., 2016). In high-resource regions, an estimated 1 in every 100 individuals develop a *Campylobacter*-related illness each year. *Helicobacter* spp. can colonize the human stomach and increase the risk of ulcers and stomach cancer (Salama et al., 2013). *Helicobacter pylori* is the most common species with some reports indicating up to 50% of the population are infected (Brown, 2000). Both *Campylobacter* spp. and *Helicobacter* spp. possess a plethora of survival and virulence factors that have allowed them to survive and persist successfully (Gundogdu et al., 2016; Hathroubi et al., 2018; Capurro et al., 2019; El Abbar et al., 2019; Liaw et al., 2019). For both *Campylobacter* spp. and *Helicobacter* spp., contaminated foods play an important role in the transmission of the microorganism to humans (Tegtmeyer et al., 2017; Ijaz et al., 2018; Quaglia and Dambrosio, 2018; Sibanda et al., 2018; McKenna et al., 2020).

The 20th International workshop on *Campylobacter, Helicobacter*, and Related Organisms (CHRO) was held in Belfast, Northern Ireland from September 8–11th, 2019. This biennial conference provided researchers with an opportunity to display the most recent findings in our understanding of *Campylobacter, Helicobacter*, and related organisms. The conference showcased the research from different topics ranging from pathogenicity and virulence factors; poultry and non-poultry epidemiology and ecology; emerging and related species; control strategies; outbreak/epidemiology and public health; detection methods and characterization; antibiotics and antimicrobials; bioinformatics, and genomics and evolution; immunology and host response. This Frontiers Research Topic provides a framework to showcase a selection of this current research.

A number of manuscripts focussed on the association between *Campylobacter* and poultry, indicating the growing importance of this research field. Quyen et al. described an optimized Loop Mediated Isothermal Amplification (LAMP) method for rapid detection of *Campylobacter* spp. in broilers, with increased specificity and sensitivity. Lafontaine et al. investigated the prebiotic galacto-oligosaccharide (GOS) on broiler chickens colonized with *C. jejuni*. The authors identified that GOS-fed birds had increased growth performance, however an increased IL-17A did not prevent colonization with *C. jejuni*. Chinivasagam et al. investigated the use of bacteriophages to control *Campylobacter* in commercially farmed broiler chickens in Australia. Muhandiramlage et al. investigated the physiological and morphological changes on *Campylobacter* isolates from chicken meat that were induced with chlorine.

Studies also continued to investigate the genomics and epidemiology of strains from different sources around the globe. Abd El-Hamid et al. described the genetic diversity of *C. jejuni* strains isolated from avian and human sources from Egypt. Terefe et al. investigated the co-occurrence of *Campylobacter* spp. in children from eastern Ethiopia and their association with environmental enteric dysfunction, diarrhea, and host microbiome. The authors highlighted the association between specific microbiome composition and gut permeability, gut inflammation, enteric dysfunction severity, and diarrhea. Mutschall et al. investigated *C. jejuni* strain dynamics in a raccoon population in southern Ontario, Canada. The authors noted that due to a high prevalence and rapid subtype turnover, racoons may act as vectors in the transmission of clinically relevant *C. jejuni* subtypes at the interface of rural, urban, and more natural environments. Hetman et al. described recovery bias of common *C. jejuni* subtypes in mixed cultures. The authors emphasized the importance of selecting multiple colonies per sample, using both enrichment and non-enrichment isolation procedures to maximize the probability of recovering multiple subtypes present in a sample. Phung et al. discussed the routes of infection of *C. hepaticus* which causes spotty liver disease in chickens. The authors highlighted that environmental sources are a likely transmission source of *C. hepaticus*.

In relation to survival, Riedel et al. analyzed the transcriptomic differences in *C. jejuni* and *C. coli* when exposed to elevated temperatures of 46°C, identifying several chaperones with increased gene expression indicative of a general involvement within heat stress response. Lv et al. described methods to detect and quantify *C. jejuni* from the viable but non-culturable (VBNC) state. The authors discuss the use of PMA-qPCR as a rapid, specific and sensitive method for the detection and quantification of VBNC *C. jejuni*.

Research focussing on immunology and host response was presented by Butkevych et al. who discussed the impact of epithelial apoptosis and subepithelial immune responses in *C. jejuni*-induced barrier disruption. The authors highlighted that *C. jejuni* infection and the consequent subepithelial immune activation leads to intestinal barrier dysfunction predominantly through caspase-3-dependent epithelial apoptosis. Pathogenicity and virulence factors were investigated by Li et al. who describe a putative novel role for FlhF in terms of directly regulating flagellar genes and further our understanding of FlhF in relation to *Campylobacter* flagellar biosynthesis and flagellation. Konkel et al. provided a comprehensive review of *Campylobacter* adherence and invasion, specifically focussing on fibronectin and binding from CadF and FlpA adhesins. Guérin et al. investigated the membrane proteocomplexome of *C. jejuni* using 2-D blue native/SDS-PAGE in conjunction with bioinformatic analysis. The authors identified a range of membrane protein complexes (MCPs) in *C. jejuni* 81–176 where these MCPs are involved in protein folding, molecules trafficking, oxidative phosphorylation, membrane structuration, peptidoglycan biosynthesis, motility and chemotaxis, stress signaling, efflux pumps, and virulence. Duma et al. discussed the influence of protein glycosylation on *C. fetus* physiology. The authors used label-free quantitative (LFQ) proteomics, identifying more than 100 proteins significantly altered in expression in two *C. fetus* subsp. *fetus* protein glycosylation (*pgl*) mutants (*pglX* and *pglJ*) compared to the wild-type strain. The authors provided a study which gives insight into the unique protein N-glycosylation pathway of *C. fetus*, but also expands our knowledge on the influence of protein N-glycosylation on *Campylobacter* cell physiology. Tejera et al. performed a genome-scale metabolic model driven design of a medium for *C. jejuni* M1cam strain. The authors showed that with a well-curated metabolic model, it is possible to design media to grow *Campylobacter* and that this has implications for the study of new *Campylobacter* species defined through metagenomics.

This Research Topic will increase the knowledge base and understanding of the processes of survival of *Campylobacter* spp. and *Helicobacter* spp. within the environment, in particular, relating to food safety, and to host-pathogen interactions.

## Author Contributions

All authors contributed to the drafting of the editorial.

## Conflict of Interest

The authors declare that the research was conducted in the absence of any commercial or financial relationships that could be construed as a potential conflict of interest.
